# Capsule deformation of a self‑expandable transcatheter aortic valve caused by repeated valve captures

**DOI:** 10.1007/s12928-026-01270-6

**Published:** 2026-03-13

**Authors:** Satoshi Kawaguchi, Yuya Kitani, Akiho Minoshima, Shingo Kunioka, Hiroyuki Kamiya, Naoki Nakagawa

**Affiliations:** 1 Department of Cardiology and Nephrology, Department of Internal Medicine, Asahikawa Medical University, Midorigaoka-Higashi 2-1-1-1, Asahikawa, 078-8510 Hokkaido, Japan; 2 Department of Emergency Medicine, Asahikawa Medical University, Asahikawa, Japan; 3 Department of Cardiac Surgery, Asahikawa medical University, Asahikawa, Japan

**Fig. 1:**
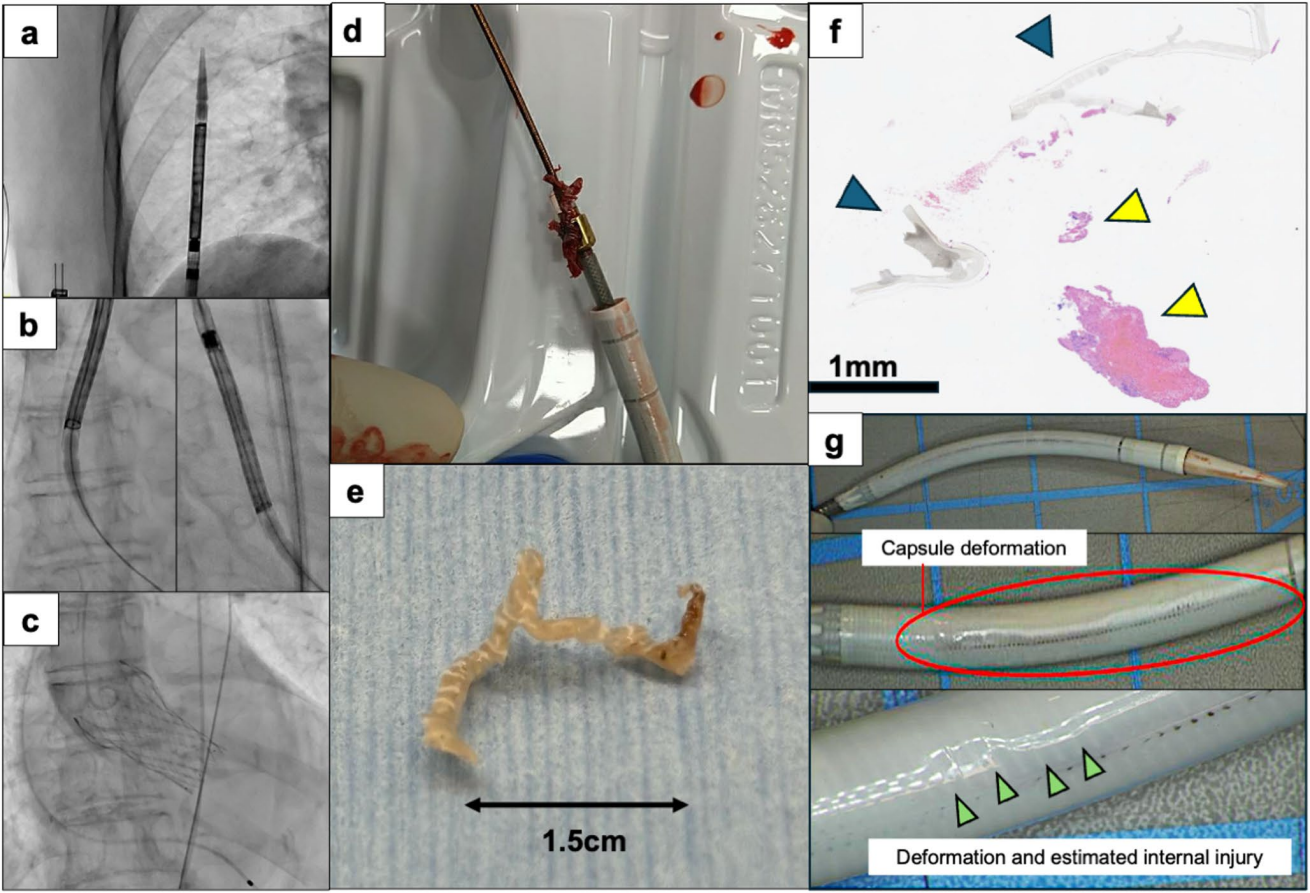
(**a**) The Evolut FX valve was correctly loaded in the capsule before insertion. (**b**) The capsule was slightly bent after the second recapture. (**c**) Final ascending aorta angiography revealed no significant aortic regurgitation. (**d**) Large debris was entwined at the paddle and valve loading position. (**e**) The debris was consist entirely of artificial objects. (**f**) Pathological analysis revealed that the debris was consist of artificial objects (blue arrows). In addition, torn pieces of aortic valve tissues were also found around the artifacts (yellow arrows). (**g**) Evident capsule deformation (red oval) and estimated internal injury (green arrows) of the Evolut FX system.

A 92-year-old woman was hospitalized for the treatment of severe aortic stenosis (AS). Our cardiovascular team considered her a candidate for transcatheter aortic valve implantation (TAVI). Contrast-enhanced multidetector computed tomography revealed an aortic annulus area of 384 mm^2^ and perimeter of 70.2 mm, for which a 26-mm self‑expandable transcatheter aortic valve (Evolut FX, Medtronic, Minesota, USA) was selected for implantation. A 14 F sheath (GORE medical, Arizona, USA) was smoothly inserted into the right femoral artery, and a 4 F sheath (Terumo Corporation, Tokyo, Japan) was introduced to the left femoral artery for pigtail catheter placement and contrast injection. The Evolut valve was correctly loaded in the capsule (Fig. 1a). Despite easy navigation of the valve delivery catheter to the aortic valve, the aorta’s horizontal angle and severe calcification of the aortic valve made it difficult to maintain co-axiality of the valve against the ascending aorta, which required repeated valve captures for accurate positioning. Although the valve capsule appeared slightly bent just after the second recapture (Fig. 1b), the valve was successfully implanted. Echocardiography confirmed no paraventricular leak and enough valve expansion. Final ascending aorta angiography showed no significant aortic regurgitation (Fig. 1c). However, on examination of the valve delivery system after TAVI, large debris was found at the paddle and valve loading position (Fig. 1d and e). Pathological examination identified that the debris was consist entirely of artificial objects and torn pieces of the patient’s aortic valve tissues were found around the objects (Fig. 1f). Analysis from Medtronic’s main office revealed that the artificial objects was derived from a part of the capsule (Fig. 1g). Based on capsule deformation, the valve or paddle may interfere with the inside of the capsule at the time of third valve deployment. Fortunately, the patient was discharged without any complications.

As far as we know, this is the first case that valve recaptures caused capsule deformation and its internal injury, which may lead to embolic events. In fact, there are no reports about the association between capsule deformation and embolic events. However, embolic events from uncertain origins during intravascular therapies may be derived from the devices. This case raises awareness regarding the risk that valve recapture may lead to capsule deformation and iatrogenic embolism.

